# Maternal Anemia and Offspring Outcomes in India: A Scoping Review

**DOI:** 10.1155/anem/2850956

**Published:** 2025-10-16

**Authors:** Melissa T. Benavente, Nophar Geifman, Sarah Bath, Anand Ahankari

**Affiliations:** ^1^ Faculty of Health and Medical Sciences, University of Surrey, Guildford, UK, surrey.ac.uk; ^2^ Faculty of Health, Psychology and Social Care, Manchester Metropolitan University, Manchester, UK, mmu.ac.uk

**Keywords:** Adolescent, child, cohort study, hemoglobin, prevalence

## Abstract

**Background:**

Anemia is a global burden, with women and children being most at risk. Moreover, anemia during pregnancy has been reported to be associated with adverse outcomes for both the mother and the offspring. In India, anemia is considered a national health issue and is estimated to have the highest prevalence in the world.

**Objectives:**

To report on the prevalence of anemia in pregnant women in India, to determine offspring outcomes that have been assessed for an association with maternal anemia in India, and to identify gaps in current knowledge.

**Methods:**

Four databases (Scopus, MEDLINE, Embase, and Web of Science) were searched using a systematic search strategy to identify relevant studies conducted in India. The search was limited to studies published between 1990 and 2023 and written in English. The exposure of interest was maternal anemia. The review was reported according to the Preferred Reporting Items for Systematic reviews and Meta‐Analyses extension for Scoping Reviews.

**Results:**

A total of 15 studies were included in this review. The reported prevalence of maternal anemia ranged from 21.26% to 90.46%. A total of 37 offspring outcomes were assessed for an association with maternal anemia, and the most widely reported outcomes related to offspring birthweight.

**Conclusion:**

Maternal anemia during pregnancy appears to adversely affect the physical and cognitive development of Indian children. However, it remains unclear as to whether such adverse effects persist into adolescence and adulthood. Further research is needed to assess the long‐term effects of maternal anemia to develop suitable health interventions.

## 1. Introduction

Anemia is a global burden affecting approximately two billion people worldwide [1]. Although prevalent in both developed and developing countries, the prevalence remains consistently higher in developing countries [2]. Women and children are those most at risk of developing anemia [2]; the global anemia prevalence is estimated to be 31% (95% confidence interval [CI]: 29–34) in women aged 15–49 years and increases to 41% (95% CI: 39–43) in pregnant women in the same age group [3]. In addition, the prevalence of anemia in pregnant women appears to be increasing worldwide [4]; a major challenge considering the Global Nutrition Target, set by the World Health Organization (WHO), for a 50% reduction in anemia prevalence within women of reproductive age by 2030 [3].

Anemia is determined by hemoglobin (Hb) concentration in the blood [5]. The WHO provides anemia thresholds, which differ according to sex, pregnancy status, age, smoking, and altitude [6]. The thresholds for pregnant women and children are listed in Table 1. These thresholds can be further expanded to specify the severity of anemia, and in pregnant women are defined as follows: mild (Hb < 11.0 g/dL), moderate (Hb < 10.0 g/dL), or severe anemia (Hb < 7.0 g/dL) [6].

**Table 1:** WHO hemoglobin thresholds (g/dL) for anemia definitions [6].

Life stage	Anemia definition using hemoglobin concentration (g/dL)
Pregnant women (trimester)	
First	< 11.0
Second	< 10.5
Third	< 11.0
Children	
Age 6–23 months	< 10.5
Age 24–59 months	< 11.0
Age 5–11 years	< 11.5
Age 12–14 years (nonpregnant girls)	< 12.0
Age 12–14 years (boys)	< 12.0
Adults	
Age 15–65 years (nonpregnant women)	< 12.0
Age 15–65 years (men)	< 13.0

The causes of anemia are often multifactorial, although the most widely reported cause is iron deficiency, with iron‐deficiency anemia (IDA) reported as the most common form of anemia [7]. Other causes of anemia include micronutrient (vitamin B12 and folate) deficiencies, genetic hemoglobinopathies, and exposure to disease(s) and infections [8].

Anemia during pregnancy is widely reported as a risk factor for a number of adverse maternal outcomes, such as preterm delivery and postpartum hemorrhage [3, 9]. In addition, maternal anemia may lead to low birth weight (LBW) and offspring mortality [3, 9, 10], as well as developmental difficulties in the offspring, including neurocognitive outcomes [2, 5] and delayed psychomotor and mental development [11]. Compromised offspring development may in turn lead to an intergenerational cycle of stunted growth and poor developmental outcomes [12].

In India, anemia is a national public health issue, and it is estimated to have the highest prevalence of anemia in the world, which is constantly increasing [8]. The National Family Health Survey (NFHS), the largest survey detailing the anemia status in India, has reported an increase in prevalence for nonpregnant and pregnant women of reproductive age (15–49 years) and children from the 2015–2016 survey to the 2019–2021 survey [13]. This is despite the implementation of several anemia prevention and treatment programs, such as iron supplementation initiatives, deworming, and screening and treatment of nonnutritional causes of anemia [14].

The purpose of this review is to gain an understanding of the research conducted in India over the past 3 decades to help address the following three aims.

⁃ To examine and report the prevalence of anemia in pregnant women across India.
⁃ To determine offspring outcomes assessed for an association with maternal anemia in India, including anemia severity and the trimester at which anemia is measured.
⁃ To identify gaps in current knowledge and areas to inform future research and public health interventions in India and other developing countries.

## 2. Methods

### 2.1. Search Strategy

The search was conducted in four electronic databases: Scopus, MEDLINE (EBSCOhost), Embase (Ovid), and Web of Science Core Collection. The search strategy focused on identifying articles reporting maternal anemia as an exposure in relation to offspring outcome(s) following live birth. All searches were limited to articles published from 1990 onwards (as of January 23, 2023) and written in English. The full search strategy for each database is given in S1.

### 2.2. Study Selection

**Figure 1:**
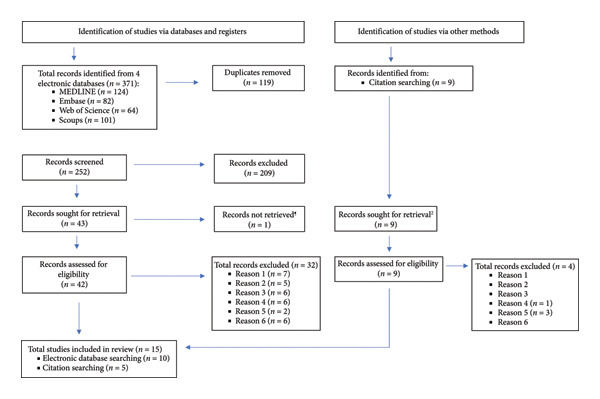
PRISMA flowchart of the search results and the selection of studies for this review. Reasons for exclusion included: (1) wrong study design; (2) maternal anemia not included as an exposure; (3) required outcomes not reported; (4) retrospective cohort study; (5) wrong study design; (6) data not reported. PRISMA flowchart adapted from Page MJ, McKenzie JE, Bossuyt PM, et al. (2021) The PRISMA 2020 statement: An updated guideline for reporting systematic reviews. BMJ 372. ^¶^Full text could not be obtained for these records.

**Figure 2:**
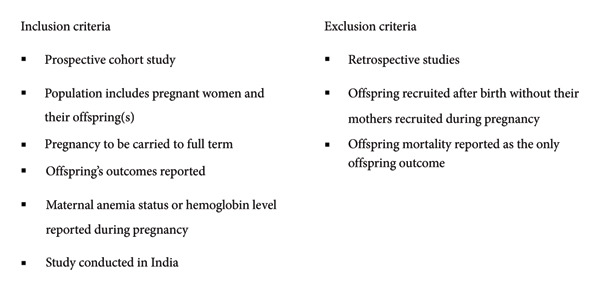
Eligibility criteria.

A total of 371 articles were retrieved from database searches. Following retrieval, duplicates were removed, and articles were assessed for eligibility using title and abstract, followed by full‐text review for those shortlisted following abstract screening (Figure 1). The inclusion and exclusion criteria are listed in Figure 2. Citation screening was undertaken on the included articles.

### 2.3. Data Extraction and Reporting

The following information was extracted from each study: author(s), year, sample size, location, anemia measurement method, anemia definition, trimester at which anemia status was recorded, reported offspring outcomes, follow‐up length, anemia prevalence, and summary of results. Definitions of the outcome variables are given in Table S2. Findings are reported according to the Preferred Reporting Items for Systematic reviews and Meta‐Analyses extension for Scoping Reviews (PRISMA‐ScR) checklist (Table S3) [15].

## 3. Results

### 3.1. Study Characteristics

A total of 15 articles were included in this review (Figure 1). All articles included here were prospective cohort studies conducted in India and published between 1990 and 2023. Thirteen studies used anemia definitions in line with those of the WHO [16–28], with three of those including trimester‐specific anemia definitions [17, 22, 27]. Seven studies reported measuring maternal Hb level during more than one trimester, although only five studies assessed outcomes in relation to this [17, 19, 22, 24, 28]. Six studies were conducted in Maharashtra state [22–26, 29], three in Karnataka state [17, 19, 27], two in Delhi city [16, 18], one each in Assam state [20], Tamil Nadu state [21], and Bihar state [28], and one study included participants from two states (Maharashtra and Karnataka) [30]. It is important to note that two of the studies are from the same cohort of participants, with each study undertaking a different analysis [25, 26]. The studies had sample sizes ranging from 211 [22] to 72,750 [24]. The follow‐up periods across studies were relatively short, with only two studies following up with the participants for longer than a year [21, 28] (Table 2).

**Table 2:** Study characteristics.

Author (year)	Location	Sample size^1^	Anemia measurement	Anemia definition/cutoff (s) (Hb in g/dL)	Time of anemia measurement	Offspring outcome (s)^2^	Follow‐up > 1 year
Hirve, S., et al. (1994)	Maharashtra	1922	Photocolorimetric	No definition for normal or standard anemia Severe (Hb < 9)	First trimester Second trimester Third trimester	LBW	N

Malhotra, M., et al. (2002)	Delhi	447	Cyanmethemoglobin	Normal (Hb ≥ 11.0) Mild (Hb 9.0–10.9) Moderate (Hb 7.0–8.9) Severe (Hb < 7.0)	Not reported	Mean birth weight, LBW, IUGR, stillbirth, neonatal death, Apgar, birth asphyxia, infectious complications	N

Shobeiri, F., et al. (2006)	Karnataka	500	Cyanmethemoglobin (finger prick method on Whatman No. 1 filter paper)	First and third trimesters Normal (Hb ≥ 11.0) Mild (Hb 9.0–10.9) Moderate (Hb 7.0–8.9) Severe (Hb < 7.0) Second trimester Normal (Hb ≥ 10.5) Mild (Hb 8.5–10.4) Moderate (Hb 6.5–8.4) Severe (Hb < 6.5)	First trimester Second trimester Third trimester	Mean birth weight, LBW	N

Kumar, A., et al. (2010)	Delhi	2027	—	Normal (Hb > 10) Mild‐to‐moderate (Hb 7.0–10) Severe (Hb < 7.0)	First trimester	LBW, birth weight, gestational age	N

Metgud, C., et al. (2012)	Karnataka	1138	—	No definition for normal or standard anemia Severe (Hb < 7.0)	First trimester Third trimester (36–37 weeks)	LBW	N

Bora R., et al. (2013)	Assam	565	—	Anemia (Hb < 11.0) Mild (Hb 10.0–10.9) Moderate (Hb 7.1–9.9) Severe (Hb < 7.0)	Delivery	GA, BW, BW percentile, chest and head circumferences, length, Apgar, birth asphyxia, respiratory distress, sepsis, hypoglycaemia, death	N

Kattula, D., et al. (2014)	Tamil Nadu	561	—	Moderate/severe (Hb < 10.0)	Post‐delivery from antenatal cards/records	LBW, overall morbidity	Y

Menon, K., et al. (2016)	Maharashtra	389	Fasting (venous blood)—method used not reported	Second trimester (Hb < 10.5) Third trimester (Hb < 11.0)	Second trimester (13–22 weeks) Third trimester (29–42 weeks) Postpartum	Weight‐for‐age, length‐for‐age, weight‐for‐length, head circumference‐for‐age, NBAS (abnormal reflex, habituation, orientation, range of state, regulation of state, motor maturity, autonomic stability)	N

Ahankari, A., et al. (2017)	Maharashtra	287	Sysmex XP‐100 automated analyzer (venous blood)	Anemia (Hb < 11) Mild (Hb 10.0–10.9) Moderate (Hb 7.9–9.9) Severe (Hb < 7.0)	First trimester to second trimester^∗^	LBW, BW	N

Patel, A., et al. (2018)	Maharashtra	72,750	Sahli’s method	Normal (Hb > 11.0) Mild (Hb 10.0–11.0) Moderate/severe (Hb < 10.0)	Varying trimesters	Stillbirth, neonatal death, LBW	N

Salunkhe A., et al. (2018)	Maharashtra	380	Laboratory parameter of Hb	Anemia (Hb < 11.0) Severe (Hb < 7.0)	First trimester	LBW, mean birth weight	N

Salunkhe, A., et al. (2019)	Maharashtra	380	Laboratory parameter of Hb	Anemia (Hb < 11.0) First trimester (Hb < 11)	First trimester	LBW	N

Finkelstein J. L., et al. (2020)	Karnataka	366	Automated Coulter counter (venous blood)	First trimester (Hb < 11.0) Second trimester (Hb < 10.5) Third trimester (Hb < 11.0)	Enrollment (< 14 weeks) Second trimester Third trimester	BW, LBW, GA at birth, preterm, SGA, Hb, anemia, length, length‐for‐age *z*‐score, stunting, weight‐for‐age z‐score, underweight, weight‐for‐length z‐score, wasting, BMI *z*‐score, thinness, ponderal index, head, chest, waist, hip circumferences, MUAC, biceps, and triceps skinfold	N

Heesemann, E., et al. (2021)	Bihar	941	HemoCue 301 device (finger‐prick)	Anemia (Hb < 11.0) Mild (Hb 10.0–10.9) Moderate (Hb 7.0–9.9) Severe (Hb < 7.0)	Varying trimesters (most in the second trimester)	Stunting, wasting, underweight, diarrhea, respiratory disease/fever, Hb, FREDI (motor skills, language skills, cognition skills, socioemotional skills)	Y

Jessani, S., et al. (2021)	Maharashtra and Karnataka	2046 (Maharashtra) 2650 (Karnataka)	HemoCue	Hb < 7.0 Hb 7.0–8.9 Hb 9.0–10.9 Hb 11.0–12.9 Hb > 13.0	First trimester Third trimester (26–30 weeks)	SGA, LBW	N

*Note:* FREDI: Frühkindliche Entwicklungsdiagnostik für Kinder von 0‐3 Jahren.

Abbreviations: GA, gestational age; IUGR, intra‐uterine growth retardation; LBW, low birth weight; MUAC, mid‐upper arm circumference; NBAS, neonatal behavioral assessment scale; SGA, small for gestational age.

^1^Sample size is the number of women recruited to each study.

^2^Outcomes included are only those which were reported in relation to the exposure to maternal anemia status or maternal hemoglobin level.

^∗^Hemoglobin measurements taken in third to fifth months of pregnancy.

### 3.2. Prevalence of Anemia in India

**Figure 3:**
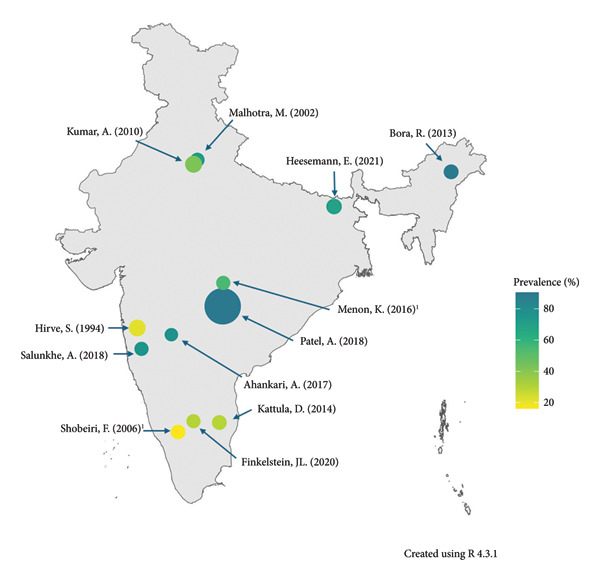
Overall anemia prevalence of included studies. Studies conducted in rural areas: Hirve (1994), Bora (2013), Menon (2016), Heesemann (2021), Patel (2018), Ahankari (2017), Salunkhe (2018). Studies conducted in urban areas: Kumar (2010), Kattula (2014), Finkelstein (2020), Malhotra (2002), and Shobeiri (2006). ^1^Third trimester prevalence.

Anemia prevalence was reported in all but three studies [19, 29, 30]; however, two studies provided enough information for prevalence to be calculated [29, 30] (Table 3). The prevalence of anemia ranged from 21.26% to 90.46% [24, 29] (Figure 3). The two studies with the highest and lowest prevalence were conducted in the Maharashtra state, although over 20 years apart, with the highest prevalence being reported more recently in 2018 [24]. Multiple studies have also been conducted in Karnataka and Delhi, and the overall prevalence for these two states was 30%–31.89% and 42.5%–72.5%, respectively [16–19]. Alongside the overall estimate of prevalence, four studies provided additional estimates for the prevalence based on anemia severity [16, 18, 20, 26], while two studies provided anemia estimates for specific trimesters [17, 22]. Of the three studies that reported prevalence by anemia severity, the severe anemia group was found to have the lowest prevalence [16, 18, 20], ranging from 0.7% [18] up to 8.3% [20]. Of the two studies reporting trimester‐specific anemia prevalence, one had measures in each trimester and found prevalence to be highest in the second trimester (49.4%) [17], while the second study found that anemia prevalence increased from 41.0% in the second trimester to 55.0% in the third trimester (no measures in the first trimester) [22].

**Table 3:** Summary of reported offspring outcomes assessed for an association with maternal anemia or hemoglobin level.

Authors	Location	Anemia prevalence (%)	Summary of results
Hirve, S., et al. (1994)	Maharashtra	21.3^∗^	LBW (maternal anemia vs. no anemia) CRR = 1.53 (95% CI: 1.10–2.10, *p* = not reported), AR = 34.5%

Malhotra, M., et al. (2002)	Delhi	Overall (72.5) Mild (47.8), moderate (17.6), severe (6.9)	LBW (maternal anemia vs. Hb 9.6–10.5 g/dL) AOR = 10.5 (95% CI: 4.5–24.5, *p* = not reported) when Hb ≤ 7.5 g/dL AOR = 4.4 (95% CI: 2.07, 9.43, *p* = not reported) when 7.6 ≤ Hb ≤ 8.5 AOR = 1.1 (95% CI: 0.54, 2.31, *p* = not reported) when 8.6 ≤ Hb ≤ 9.5 AOR = 1.6 (95% CI: 0.76, 3.36, *p* = not reported) when 10.6 ≤ Hb ≤ 11.5 AOR = 5.2 (95% CI: 2.39–116, *p* = not reported) when Hb ≥ 11.6 g/dL OR = 0.86 (95% CI: 0.76–0.97, *p* = not reported) Highest mean birth weight (g) 2807 ± 511.0 when 9.6 ≤ Hb ≤ 10.5 g/dL Apgar score < 8 7.5% prevalence when 7.0 ≤ Hb ≤ 8.9 g/dL 25.8% prevalence when Hb ≤ 7.0 g/dL

Shobeiri, F., et al. (2006)	Karnataka	45.6 (1st trimester) 49.4 (2nd trimester) 16.2 (3rd trimester)	LBW (maternal anemia vs. no anemia) RR = 2.13 (95% CI: not reported, *p* = not reported) when anemic in the first trimester RR = 1.54 (95% CI: not reported, *p* = not reported) when anemia in the second trimester RR = 1.89 (95% CI: not reported, *p* = not reported) when anemic in the third trimester Highest mean birth weight (g) 3094 when anemic status is normal for all three trimesters in the high‐income group

Kumar, A., et al. (2010)	Delhi	Overall (42.5) Mild/moderate (41.8) Severe (0.7)	LBW (maternal Hb ≤ 10.0 g/dL vs. maternal Hb ≥ 10.0 g/dL) OR = 1.02 (95% CI: 0.83–1.25, *p* = 0.830) Birth weight *β* = 0.00 (95% CI: not reported, *p* = 0.984) Gestational age *β* = 0.04 (95% CI: not reported, *p* = 0.069)

Metgud, C., et al. (2012)	Karnataka	—	LBW “When compared with mothers who had severe anemia in the first trimester, the risk of LBW increased from 5 to 20 times.” RR = not reported (95% CI: not reported, *p* = not reported)

Bora R., et al. (2013)	Assam	Overall (89.6) Mild (21.6), moderate (70.1), severe (8.3)	Mild anemia (maternal anemia vs. no anemia) Gestational age (weeks) Mean difference = −0.05 (95% CI: −0.38–0.28, *p* = 0.78) Birth weight (g) Mean difference = −153.60 (95% CI: −291.40 to −15.69, *p* = 0.03) Birth weight percentile Mean difference = −7.55 (95% CI: −15.82–0.72, *p* = 0.07) Chest circumference (cm) Mean difference = −0.79 (95% CI: −1.22 to −0.36, *p* < 0.001) Head circumference (cm) Mean difference = −0.59 (95% CI: −1.04 to −0.13, *p* = 0.01) Length (cm) Mean difference = −1.26 (95% CI: −2.29 to −0.23, *p* = 0.02) Apgar, 1 min Mean difference = −0.58 (95% CI: −0.92 to −0.23, *p* = 0.01) SGA OR = 1.23 (95% CI: 0.36–4.26, *p* = 0.74) Moderate anemia (maternal anemia vs. no anemia) Gestational age (weeks) Mean difference = −0.38 (95% CI: −0.66–0.11, *p* = 0.01) Birth weight (g) Mean difference = −88.49 (95% CI: −206.30–29.30, *p* = 0.14) Birth weight percentile Mean difference = −0.88 (95% CI: −8.31–6.56, *p* 0.81) Chest circumference (cm) Mean difference = −0.49 (95% CI: −0.84 to −0.15, *p* = 0.01) Head circumference (cm) Mean difference = −0.03 (95% CI: −0.38–0.31, *p* = 0.86) Length (cm) Mean difference = −0.19 (95% CI: −0.75–0.37, *p* = 0.50) Apgar, 1 min Mean difference = −0.65 (95% CI: −0.91 to −0.40, *p* < 0.001) SGA OR = 1.16 (95% CI: 0.67–2.00, *p* = 0.59) Severe anemia (maternal anemia vs. no anemia) Gestational age (weeks) Mean difference = −0.63 (95% CI: −1.23 to −0.03, *p* = 0.04) Birth weight (g) Mean difference = −481.70 (95% CI: −658.30 to −305.00, *p* < 0.001) Birth weight percentile Mean difference = −21.14 (95% CI: −29.86 to −12.42, *p* < 0.001) Chest circumference (cm) Mean difference = −1.80 (95% CI: −2.47 to −1.13, *p* < 0.001) Head circumference (cm) Mean difference = −1.03 (95% CI: −1.64 to −0.43, *p* = 0.001) Length (cm) Mean difference = −1.67 (95% CI: −2.52 to −0.82, *p* < 0.001) Apgar, 1 min Mean difference = −0.73 (95% CI: −1.04 to −0.42, *p* < 0.001) SGA OR = 1.89 (95% CI: 1.25–2.86, *p* + 0.002)

Kattula, D., et al. (2014)	Tamil Nadu	29.6 (Hb < 10 g/dL), 57.4 (Hb 10–12 g/dL)	LBW (maternal anemia (Hb < 10 g/dL) vs. no anemia) COR = 2.0 (95% CI: 0.98–4.06, *p* = 0.05) AOR = 2.36 (95% CI: 1.08–5.18, *p* = 0.03) Overall morbidity Crude HR: 0.99 (95% CI: 0.86–1.14, = 0.93)

Menon, K., et al. (2016)	Maharashtra	41.0 (2nd trimester) 55.0 (3rd trimester)	Second trimester (maternal anemia vs. no anemia) Weight‐for‐age *z*‐score Crude mean difference = 0.19 (95% CI: −0.04–0.43, *p* = 0.108) Adjusted mean difference = 0.26 (95% CI: 0.03–0.50, *p* = 0.029) Length‐for‐age *z*‐score Crude mean difference = 0.40 (95% CI: 0.11–0.69, *p* = 0.008) Adjusted mean difference = 0.50 (95% CI: 0.20–0.79, *p* = 0.001) Weight‐for‐length *z*‐score Crude mean difference = −0.20 (95% CI: −0.51–0.11, *p* = 0.195) Adjusted mean difference = −0.19 (95% CI: −0.51–0.14, *p* = 0.255) Head circumference‐for‐age *z*‐score Crude mean difference = 0.21 (95% CI: −0.01–0.42, *p* = 0.061) Adjusted mean difference = 0.26 (95% CI: 0.03–0.49, *p* = 0.029) Abnormal reflex Crude mean difference = −0.34 (95% CI: −1.79–1.10, *p* = 0.642) Adjusted mean difference = −0.70 (95% CI: −2.18 to 0.78, *p* = 0.350) Habituation Crude mean difference = 0.06 (95% CI: −1.10–1.21, *p* = 0.919) Adjusted mean difference = 0.18 (95% CI: −1.03–1.39, *p* = 0.772) Orientation Crude mean difference = 0.89 (95% CI: −1.77–3.55, *p* = 0.508) Adjusted mean difference = 1.16 (95% CI: −1.66–3.98, *p* = 0.417) Range of state Crude mean difference = −0.82 (95% CI: −1.52 to −0.13, *p* = 0.021) Adjusted mean difference = −0.83 (95% CI: −1.56 to −0.09, *p* = 0.029) Regulation of state Crude mean difference = −0.57 (95% CI; −2.09–0.95, *p* = 0.463) Adjusted mean difference = −0.30 (95% CI: −1.97–1.37, *p* = 0.720) Motor maturity Crude mean difference = −0.76 (95% CI: −1.84–0.31, *p* = 0.162) Adjusted mean difference = −0.41 (95% CI: −1.54–0.72, *p* = 0.475) Autonomic stability Crude mean difference = 1.17 (95% CI: −0.32–2.66, *p* = 0.121) Adjusted mean difference = 1.35 (95% CI: −0.47–3.18, *p* = 0.143) Third trimester (reference no anemia) Weight‐for‐age Crude mean difference = 0.13 (95% CI: −0.11–0.36, *p* = 0.286) Adjusted mean difference = 0.17 (95% CI: 0.06–0.41, *p* = 0.152) Length‐for‐age Crude mean difference = 0.18 (95% CI: −0.11–0.47, *p* = 0.217) Adjusted mean difference = 0.26 (95% CI: −0.04–0.56, *p* = 0.087) Weight‐for‐length Crude mean difference = −0.05 (95% CI: −0.36–0.27, *p* = 0.778) Adjusted mean difference = 0.08 (95% CI: −0.41–0.25, *p* = 0.624) Head circumference‐for‐age Crude mean difference = 0.17 (95% CI: −0.04–0.38, 0.117) Adjusted mean difference = 0.16 (95% CI: −0.07–0.39, *p* = 0.180) Abnormal reflex Crude mean difference = −0.72 (95% CI: −2.15–0.70, *p* = 0.317) Adjusted mean difference = −1.43 (95% CI: −2.90–0.04, *p* = 0.057) Habituation Crude mean difference = −0.21 (95% CI: −1.30–0.88, *p* = 0.70) Adjusted mean difference = −0.13 (95% CI: −1.29–1.02, *p* = 0.818) Orientation Crude mean difference = 3.58 (95% CI: 1.08–6.08, *p* = 0.005) Adjusted mean difference = 3.88 (95% CI: 1.23–6.53, *p* = 0.004) Range of state Crude mean difference = 0.20 (95% CI: −0.51–0.92, *p* = 0.573) Adjusted mean difference = 0.34 (95% CI: −0.43–1.10, *p* = 0.390) Regulation of state Crude mean difference = 0.03 (95% CI: −1.45–1.52, *p* = 0.963) Adjusted mean difference = 0.23 (95% CI: −1.42–1.88, *p* = 0.782) Motor maturity Crude mean difference = 0.25 (95% CI: −0.80–1.31, *p* = 0.636) Adjusted mean difference = 0.44 (95% CI: −0.69–1.56, *p* = 0.442) Autonomic stability Crude mean difference = 0.20 (95% CI: −1.37–1.77, *p* = 0.798) Adjusted mean difference = 0.20 (95% CI: −1.68–2.08, *p* = 0.831)

Ahankari, A., et al. (2017)	Maharashtra	77.0	LBW (maternal anemia vs. no anemia) COR = 0.34 (95% CI: 0.13–0.92, *p* ≤ 0.05) Birth weight (kg) Unadjusted *β* = 0.21 (95% CI: 0.08–0.33, *p* < 0.05)

Patel, A., et al. (2018)	Maharashtra	90.46	LBW (maternal anemia vs. no anemia) Mild anemia RR = 1.09 (95% CI: 1.00–1.18, *p* = greater than 0.05) when GA at enrollment ≤ 20 weeks RR = 1.07 (95% CI: 0.96–1.20, *p* = greater than 0.05) when GA at enrollment > 20 weeks RR = 1.08 (95% CI: 1.01–1.15, *p* < 0.05) all women Severe/moderate anemia RR = 1.24 (95% CI: 1.14–1.35, *p* < 0.001) when GA at enrollment ≤ 20 weeks RR = 1.33 (95% CI: 1.19–1.49, *p* < 0.001) when GA at enrollment > 20 weeks RR = 1.26 (95% CI: 1.18–1.35, *p* < 0.001) all women

Salunkhe A., et al. (2018)	Maharashtra	Overall (75.8) Mild (43.2), moderate (28.9), severe (3.7)	Highest mean birth weight (g) ± SD (g) 2790.40 ± 443.10 (mild anemia group^¶^)

Salunkhe, A., et al. (2019)	Maharashtra	62.7 (1^st^ trimester)	LBW (maternal anemia vs. no anemia) RR = 1.24 (95% CI: 1.08–1.43, p^ǂ^) AR = 19.5%

Finkelstein J. L., et al. (2020)	Karnataka	30.0	Model 1 (maternal anemia vs. no anemia) Birthweight (g) *β* = −174.7 (SE: 61.2, *p* = 0.004) LBW RR = 1.84 (95% CI: 1.03–3.29, *p* = 0.04) Gestational age at birth (weeks) Adjusted *β* = −0.91 (SE: 0.28, *p* = 0.001) SGA RR = 1.24 (95% CI: 0.87, 1.76, *p* = 0.23) Hb (g/dL) *β* = 0.14 (SE: 0.44, *p* = 0.76) Anemia RR = 0.83 (95% CI: 0.49–1.40, *p* = 0.49) Length (cm) *β* = −0.41 (SE: 0.41, *p* = 0.31) LAZ *β* = −0.08 (SE: 0.20, *p* = 0.6) LAZ < −2 (stunting) RR = 0.96 (95% CI: 0.62, 1.50, *p* = 0.87) WAZ Adjusted *β* = −0.41 (SE: 0.14, *p* = 0.004) WAZ < −2 (underweight) ARR = 1.99 (95% CI: 1.03–3.87, *p* 0.04) WLZ *β* = −0.45 (SE: 0.27, *p* = 0.09) WLZ < −2 (wasting) RR = 1.52 (95% CI: 0.78, 2.97, *p* = 0.22) BMIZ *β* = −0.48 (SE: 0.22, *p* = 0.03) BMIZ < −2 (thinness) ARR = 2.42 (95% CI: 1.34–4.39, *p* = 0.004) Ponderal index *β* = −0.001 (SE: 0.0007, *p* = 0.17) Head circumference *β* = −0.27 (SE: 0.24, *p* = 0.24) Chest circumference *β* = −0.56 (SE: 0.29, *p* = 0.05) Waist circumference *β* = −0.61 (SE: 0.40, *p* = 0.13) Hip circumference *β* = 0.03 (SE: 0.58, *p* = 0.96) MUAC (cm) *β* = −0.94 (SE: 0.46, *p* = 0.04) Biceps skinfold *β* = −0.07 (SE: 0.14, *p* = 0.65) Triceps skinfold *β* = 0.06 (SE: 0.22, *p* = 0.80) Model 2 (maternal anemia vs. no anemia) Birthweight (g) *β* = −1.66.8 (SE: 61.1, *p* = 0.006) LBW RR = 2.15 (95% CI: 1.20–3.84, *p* = 0.01) Gestational age at birth (weeks) *β* = −0.95 (SE: 0.28, *p* = 0.0007) SGA RR = 1.25 (95% CI: 0.87–1.81, *p* = 0.23) Hb (g/dL) *β* = −0.11 (SE: 0.44, *p* = 0.80) Anemia RR = 1.02 (95% CI: 0.57–1.83, *p* = 0.95) Length (cm) *β* = −0.35 (SE: 0.41, *p* = 0.39) LAZ Β = −0.03 (SE: 0.20, *p* = 0.89) LAZ < −2 (stunting) RR = 0.85 (95% CI: 0.54–1.33, *p* = 0.47) WAZ *β* = −0.41 (SE: 0.14, *p* = 0.004) WAZ < −2 (underweight) RR = 2.20 (95% CI: 1.16–4.15, *p* = 0.02) WLZ *β* = −0.40 (SE: 0.27, *p* = 0.15) WLZ < −2 (wasting) RR = 1.50 (95% CI: 0.76–2.95, *p* = 0.25) BMIZ *β* = −0.45 (SE: 0.22, *p* = 0.04) BMIZ < −2 (thinness) RR = 2.49 (95% CI: 1.37–4.56, *p* = 0.003) Ponderal index *β* = −0.001 (SE = 0.0007, *p* = 0.18) Head circumference *β* = −0.021 (SE: 0.24, *p* = 0.38) Chest circumference *β* = −0.61 (SE: 0.30, *p* = 0.04) Waist circumference Β = −0.61 (SE: 0.41, *p* = 0.13) Hip circumference *β* = −0.06 (SE: 0.57, *p* = 0.91) MUAC (cm) *β* = −0.94 (SE: 0.45, *p* = 0.03) Biceps skinfold *β* = −0.09 (SE: 0.15, *p* = 0.54) Triceps skinfold *β* = 0.03 (SE: 0.22, *p* = 0.90)

Heesemann, E., et al. (2021)	Bihar	69.0	Model 1 (maternal Hb as exposure) Offspring Hb *β* = 0.17 (95% CI: 0.11–0.23, *p* < 0.01) Motor skills *β* = −0.01 (95% CI: −0.05–0.03, *p* = greater than 0.05) Language skills *β* = −0.01 (95% CI: −0.05–0.04, *p* = greater than 0.05) Cognition skills *β* = −0.03 (95% CI: −0.07–0.00, *p* < 0.1) Socioemotional skills *β* = −0.02 (95% CI: −0.05–0.02, *p* = greater than 0.05) Model 2 (mild maternal anemia vs. no anemia) Offspring Hb *β* = −0.20 (95% CI: −0.41–0.00, *p* < 0.1) Motor skills *β* = 0.03 (95% CI: −0.13–0.19, *p* = greater than 0.05) Language skills *β* = 0.01 (95% CI: −0.13–0.15, *p* = greater than 0.05) Cognition skills *β* = 0.06 (95% CI: −0.09–0.22, *p* = greater than 0.05) Socioemotional skills *β* = 0.05 (95% CI: −0.08–0.17, *p* = greater than 0.05) (Moderate/severe anemia vs. no anemia) Offspring Hb *β* = −0.57 (95% CI: −0.78 to −0.36, *p* < 0.001) Motor skills Β = 0.06 (95% CI: −0.10–0.22, *p* = greater than 0.05) Language skills *β* = 0.03 (95% CI: −0.12–0.19, *p* = greater than 0.05) Cognition skills *β* = 0.12 (95% CI: −0.02–0.26, *p* < 0.01) Socioemotional skills *β* = 0.01 (95% CI: −0.12–0.15, *p* = greater than 0.05)

Jessani, S., et al. (2021)	Maharashtra and Karnataka	31.89^∗^	Mild anemia LBW (maternal anemia vs. no anemia) RR = 1.07 (95% CI: 0.96–1.19, *p* = not reported) SGA RR = 1.07 (95% CI: 0.99–1.15, *p* = not reported) Moderate anemia LBW RR = 1.02 (95% CI: 0.81–1.29, *p* = not reported) SGA RR = 1.12 (95% CI: 0.96–1.30, *p* = not reported) High Hb level LBW RR = 1.13 (95% CI: 0.96–1.34, *p* = not reported) SGA RR = 1.02 (95% CI: 0.90–1.16, *p* = not reported)

*Note:* β: β coefficient from linear regression; Hb: hemoglobin; SGA: small for gestation; N/A: not applicable, as authors did not report any outcomes to be associated with the exposure of interest in the India cohort.

Abbreviations: 95% CI, 95% confidence interval; AOR, adjusted odds ratio; AR, attributable risk; CRR, crude relative risk; LBW, low birth weight; MUAC, mid‐upper arm circumference; OR, odds ratio; SD, standard deviation; SE, standard error.

^∗^Prevalence not reported but has been calculated by authors of this review using study sample size and number of anemic mothers as reported in the article.

^¶^Authors did not define Hb range.

^ǂ^Authors stated estimate is significant but have not reported significance level or *p*‐value.

### 3.3. Offspring Outcomes Associated With Maternal Anemia

Across studies, a total of 37 offspring outcomes were assessed for an association with maternal anemia (Table 2). For the purpose of this review, outcomes have been grouped into anthropometry, Apgar score, gestational age (GA), Hb, and cognitive skills.

#### 3.3.1. Anthropometry

The most widely reported anthropometric outcomes were those relating to offspring birth weight. Twelve studies investigated birth weight as an outcome (Table 3). Overall, there appears to be evidence suggesting that, when compared to nonanemic women, maternal anemia during pregnancy may adversely affect the birth weight of offspring by increasing the risk or odds of LBW [21, 24, 26, 27] and by resulting in offspring that are lighter at birth (*β* coefficient −166.8, standard error [SE]: 61.1, *p* = 0.006) [27]. In addition, when assessing anemia severity, in comparison to nonanemic women, women with mild and severe anemia had offspring with a mean birth weight 153.60 g (*β*: −153.60, 95% CI: −291.4 to −15.69, *p* = 0.03) and 481.70 g (*β*: −481.70, 95% CI: −658.30 to −305.00, *p* < 0.001) lighter, respectively [20]. Bora et al. also report severe anemia as leading to a reduction in birth weight percentile (*β*: −21.14, 95% CI: −29.86 to −12.42, *p* < 0.001) [20]. However, the suggested association between maternal anemia and offspring birth weight was not uniform: one study reported anemic women having offspring 210 g heavier at birth compared to nonanemic women (*β*: 0.21, 95% CI: 0.08–0.33, *p* < 0.05), and as having reduced odds of an LBW baby (odds ratio [OR]: 0.34, 95% CI: 0.13–0.92, *p* = 0.03) [23].

In three studies, offspring physical growth was assessed across five indicators: weight‐for‐age (WAZ), length‐for‐age (LAZ), weight‐for‐length (WLZ) [22, 27, 28], BMI‐for‐age (BMIZ) [27], and head circumference‐for‐age [22]. The reported associations differed across indicators. Both WAZ [22, 27] and LAZ [22] were associated with maternal anemia when compared to nonanemic women. Yet the associations reported by Menon et al. were for women who were anemic in the second trimester, not in the third trimester [22]. Only one study reported BMIZ and thinness (defined as Z‐score < −2 SD of BMIZ) and found that offspring born to anemic women had lower BMIZ scores at birth (*β*: −0.45, SE: 0.22, *p* = 0.04) and over two times the risk of having offspring with thinness (RR: 2.49, 95% CI: 1.37–4.56, *p* = 0.03) when compared to nonanemic women [27]. Likewise, Menon et al., the only study to assess head circumference‐for‐age, found that nonanemic women had offspring with larger head circumference‐for‐age (SD: 0.26, 95% CI: 0.03–0.49, *p* = 0.029) when compared to women that were anemic in the second trimester [22]. None of the three studies found evidence of an association between maternal anemia and offspring WLZ.

A number of other anthropometric offspring outcomes were assessed: length [20, 27], chest circumference [20, 27], head circumference [20, 27], waist and hip circumference [27], mid‐upper arm circumference (MUAC) [27], and biceps and triceps skinfold [27]. Again, there is evidence that maternal anemia may lead to adverse physical growth in offspring with regard to length, chest circumference, head circumference, and MUAC. Both mild and severe anemia were found to be associated with a 1.26 cm (95% CI: −2.29 to −0.23, *p* = 0.02) and 1.67 cm (95% CI: −2.52 to −0.82, *p* < 0.001) smaller mean difference in offspring length, respectively, when compared to no anemia [20]. Moderate anemia, however, was not found to be associated with offspring length [20], nor was overall anemia status [27]. After adjusting for GA of sample, vitamin B12 intervention status, maternal BMI, educational level, standard of living index (SLI) > 28, and the natural log of C‐reactive protein (ln(CRP)), offspring born to anemic women had smaller chest circumferences (*β*: 0.61, SE: 0.30, *p* = 0.04) and smaller MUAC (*β*: −0.94, SE: 0.45, *p* = 0.03), when compared to nonanemic women [27]. Likewise, when comparing women with severe anemia to nonanemic women, the mean difference in chest circumference for offspring born to women with severe anemia was 1.80 cm smaller (*β*: −1.80, 95% CI: −1.13 to −2.47, *p* < 0.001) [20]. Both mild and severe anemia were found to be associated with 0.59 cm (*β*: −0.59, 95% CI: −1.04 to −0.13, *p* = 0.01) and 1.03 cm (*β*: −1.03, 95% CI: −1.64 to −0.43, *p* = 0.001) smaller head circumference, respectively, when compared to nonanemic women [20]. This association was not, however, supported by Finkelstein et al., who found neither maternal anemia nor maternal Hb to be associated with head circumference [27]. The same study investigated the effect of maternal anemia on waist and hip circumference and triceps and biceps skinfold and did not find any of these outcomes to be associated with maternal anemia during pregnancy [27].

#### 3.3.2. Apgar Score

Two studies investigated the effect of anemia severity on Apgar scores [16, 20]. When comparing the difference in scores to nonanemic women, Bora et al. found that children born to women with mild and severe anemia had Apgar scores that were lower by 0.58 (*p* = 0.01) and 0.73 (*p* < 0.001) [20]. By contrast, Malhotra et al. reported the number of offspring with Apgar scores of less than eight (*n* = 14), which did not differ significantly between anemia severity levels (Table 3).

#### 3.3.3. Gestational Age

Three studies reported GA [18, 20, 27], while three assessed small for gestational age (SGA) as an outcome [20, 27, 30] (Table 3). The studies used varying definitions for GA and SGA (Table S2). Bora and team found mean GA to be shorter by 0.38 weeks (95% CI: 0.11–0.66, *p* = 0.007) and 0.68 weeks (95% CI: 0.03–1.23, *p* = 0.04) when comparing women with moderate and severe anemia to nonanemic women, respectively. The authors also found severe maternal anemia to almost double the odds of having an SGA baby (OR: 1.89, 95% CI: 1.25–2.86, *p* = 0.002), when compared to nonanemic women [20]. This conflicts with Jessani et al. who did not find an association between varying Hb concentrations, including severe anemia, with SGA [30]. Likewise, Finkelstein et al. assessed maternal anemia and Hb concentration and did not find either to be associated with SGA [27]. By contrast, Finkelstein and colleagues found evidence of an association between maternal exposures and GA at delivery: Maternal anemia decreased the GA at delivery by 0.95 weeks (SE: 0.28, *p* = 0.0007). Additionally, the authors also found that, as maternal Hb concentration increased, GA at birth increased by 0.28 weeks (SE: 0.08, *p* = 0.001). This concurs with Bora et al. who report a decrease of 1.0 g/dL in maternal Hb was associated with a decrease in GA by 0.179 weeks (95% CI: not reported, *p* = 0.003), but differs from Kumar et al., who did not find evidence of an association between maternal Hb and GA at age (*β*: 0.04, 95% CI: not reported, *p* = 0.069).

#### 3.3.4. Hb

Both Heesemann et al. and Finkelstein et al. investigated the association between maternal Hb and offspring Hb, with contradicting results. Heesemann et al. found strong evidence of an association; after adjusting for the mother’s Hb level after pregnancy, an Hb increase of 1.0 g/dL during pregnancy was associated with an increase in offspring’s Hb by 0.17 g/dL when aged between 22 and 32 months (*β*: 0.17, 95% CI: 0.17–0.23, *p* < 0.01) (Table 3). The authors also found an association when assessing a nonlinear association; both mild anemia (*β*: −0.20, 95% CI: −0.41 to 0.00, *p* < 0.01) and moderate and severe anemia (*β*: −0.57, 95% CI: −0.78 to −0.36, *p* < 0.01) resulted in a reduction in offspring Hb when compared to nonanemic women [28]. By contrast, Finkelstein et al. did not identify any association between maternal anemia or maternal Hb with offspring Hb.

#### 3.3.5. Cognitive Skills

Heesemann et al. and Menon et al. reported associations between maternal anemia and offspring cognitive skills using an adapted version of the German development test, FREDI 0–3 [31, 32], and Neonatal Behavioral Assessment Scores (NBAS) [33], respectively (Table 3). Heesemann et al. found a positive association between both maternal Hb during pregnancy and moderate and severe anemia with offspring cognition skills, yet there is only weak evidence of an association (*p* < 0.1) [28]. Menon et al., however, found stronger evidence of an association between maternal anemia and offspring orientation cluster skills, which are reflective of a child’s social interaction capacities. The authors report that offspring of nonanemic women had orientation scores 3.88 higher, i.e. better social interaction capacities, than those born to anemic women (*p* = 0.004). This association was only reported for women who were anemic in the third trimester and did not hold true for women who were anemic in the second trimester [22]. It is important to note that both FREDI 0–3 and NBAS consist of a number of outcomes (Table 2), yet the remaining outcomes were not found to be associated with maternal anemia.

## 4. Discussion

Efforts to reduce the global anemia rate are not yet achieving targets set by the WHO, and countries are being encouraged to accelerate their efforts in reducing anemia prevalence [34]. India, having the highest prevalence of anemia in the world [8], has a key role to play regionally and internationally in meeting such targets. This scoping review of 15 cohort studies can help to better understand the current state of research related to maternal anemia specifically and its effects on offspring outcomes in India.

The studies included in this review report prevalence and outcomes associated with maternal anemia from all regions of the country. The reported prevalence of anemia varied widely across and within states. The disparities in prevalence may be attributable to a number of reasons. A variety of methods were used to determine Hb levels, including point‐of‐care devices [24, 28–30], the cyanmethemoglobin method (CM) [16, 17], and automated analyzers [23, 27]. The CM is considered the gold standard for determining Hb levels; however, it is not always appropriate in resource‐poor settings such as in developing countries. In contrast, the rapid results provided by point‐of‐case devices are widely deemed to be more subjective and less precise, so they should be interpreted with caution [35, 36]. There is limited evidence that HemoCue and other Hb measurement devices under‐ or overestimate anemia prevalence, depending on whether venous or capillary blood samples are used [35]; however, prevalence estimates based on methods of measurement did not appear to differ among included studies in our review. In addition, the studies varied between urban [16–18, 21, 27] and rural [19, 20, 22–26, 28–30] settings. In India, there is a great deal of disparity between urban and rural areas, with the NFHS 5 reporting that more than half of the rural population (54%) is considered to be in the two lowest wealth quintiles, whilst three‐quarters (75%) of the urban population is in the highest two wealth quintiles [37]. Wealth quintiles can be considered an indicator of socio‐economic status (SES), a widely reported risk factor for anemia [10, 11, 14]; such wealth disparities between urban and rural areas are likely to affect the prevalence of anemia between the two. Further, the Hb thresholds used to define anemia were not uniform across included studies. The lowest reported anemia prevalence (of 21.26%) was from the Maharashtra state and defined as Hb less than 9.0 g/dL [29], which is lower than the WHO’s trimester‐specific cutoffs [6]. This will have resulted in an underestimation of the prevalence reported by Hirve and Ganatra, as women with mild anemia and those with moderate anemia above 9.0 g/dL will not have contributed to the prevalence estimate [29].

In addition, the use of nutritional supplements may have also impacted the anemia prevalence reported in included studies. Nine of the included studies report on the use of iron supplementation during pregnancy [16, 17, 19, 22, 23, 25–28]. It is important to note that national anemia prevention strategies have been active in India in some form since 1970 [14]. Iron supplementation during pregnancy may reduce the risk of maternal anemia [38], and the WHO recommends daily iron (and folic acid) supplementation for pregnant women [39]. As such, anemia prevalences reported in this review may have been higher in the absence of iron supplementation. The disparity in maternal anemia prevalence highlighted in this review is in line with findings from two recent surveys from India, NFHS 5 and the District Level Household and Facility Survey (DLHS 4). The NFHS 5 reported the highest prevalence of anemia among pregnant women aged 15 to 49 to be 78% in the Ladakh region, while the lowest was 21% in the Lakshadweep territory [37]. Likewise, estimates from the DLHS 4 ranged from 34.6% to 79.2% in Kerala and West Bengal, respectively [40].

Such differences in prevalence have also been reported in other developing countries. Systematic reviews in South Africa and Sudan reported maternal anemia prevalence ranging from 20% to 40% and 23%–76%, respectively [10, 41]. The WHO’s 2019 estimated prevalence of anemia in pregnant women aged 15 to 49 in India was 50.1% (95% CI: 44.2%, 54.2%), which falls within the range identified in this review [42]. Such a prevalence is the highest reported in Southeast Asia and is much higher than India’s contiguous neighbors, such as Pakistan, Nepal, and Bangladesh, which have an estimated prevalence of 44% (95% CI: 31.5%–52.7%), 42.4% (95% CI: 31.4%–50.3%), and 42.2% (95% CI: 27.9%–52%), respectively [42].

Here we highlight a range of offspring outcomes that have been assessed for an association with maternal anemia and can be grouped into five categories: anthropometry, Apgar score, GA, Hb, and cognitive skills. Anthropometry outcomes were the most widely investigated [16, 18–27, 29, 30], with outcomes such as LBW, mean birth weight, birth weight percentile, length, LAZ, WAZ, BMIZ, thinness, head circumference‐for‐age, chest circumference, head circumference, and MUAC all reported as associated with maternal anemia. Although for some, contradictory associations were observed, suggesting that anemia during pregnancy may interrupt many aspects of fetal growth. For example, one study did report a protective effect of maternal anemia on LBW [23], which is contradictory to findings from other studies included in this review and the wider literature, where there is sufficient evidence supporting its adverse association with maternal anemia [3, 9, 43]. The protective effect reported may be attributable to the small number of LBW events in the study (*n* = 18) and the reporting of birthweight retrospectively from birth records via telephone interview with the mothers [23]. Using standard logistic regression with a small number of outcome events may result in small‐sample bias and underestimate the effect size [44], whilst retrospective data collection may also underestimate the effect size due to recall bias. Further research is needed, however, to confirm whether the adverse association between maternal anemia and LBW extends to other aspects of fetal growth during in‐utero development. Only two studies in this review investigated cognitive outcomes, and both reported limited evidence of an association with maternal anemia [22, 28]. There is evidence in the literature, however, that maternal anemia is associated with offspring neurodevelopment [2], adverse brain development (reduced basal ganglia and corpus callosum volume) at age 2–3 years [5], and poorer auditory brainstem response within 48 h of birth when maternal serum ferritin is < 15 μg/L [45]. Further research is needed to investigate the effects of maternal anemia on cognitive outcomes in Indian children through a life‐course approach where its associations with brain development could be studied from childhood into adulthood.

This review also reported the role of anemia severity and trimester of gestation on prevalence. The finding that only a small number of women were severely anemic aligns with the wider literature from both India and globally [4, 7, 10]. However, when comparing prevalence in the second and third trimesters, we found conflicting results. Shobeiri and colleagues reported a decrease of 33.2%, while Menon et al. reported an increase of 14% when going from the second to the third trimester [17, 22]. This difference may be due to a higher total iron intake by women during the second half of pregnancy in Shobeiri and colleagues’ study and the higher SES of women included in their study, as lower SES has been found to increase the risk of anemia [7]. The literature remains limited as to how and why anemia prevalence changes across trimesters and, more importantly, how this affects fetal growth and offspring outcomes [2, 3].

The severity of anemia was also assessed with regard to offspring outcomes. We found evidence that increased anemia severity in women during pregnancy may lead to increased risk, odds, and a greater mean difference for offspring outcomes such as LBW, chest circumference, head circumference, length, and Hb. We also found evidence that severe anemia often had a greater magnitude of effect on outcomes compared to mild or moderate anemia. Some studies have reported a U‐shaped relationship between maternal Hb levels and offspring outcomes, with both very high and low maternal Hb associated with adverse offspring outcomes [3]. This relationship is postulated to be linked to plasma volume expansion, which happens predominantly during the second and third trimesters. Such expansion is associated with lower concentrations of maternal Hb; thus, high levels of maternal Hb are believed to be a marker of poor plasma volume expansion, which itself is associated with adverse birth outcomes [3].

A key strength of this review is the systematic search strategy, which was employed across four databases (S1), and reporting in accordance with PRISMA‐ScR (Table S3). To our knowledge this review is the first where all cohort studies from India were identified and included to investigate the impact of maternal anemia on a range of child outcomes. This review also has some limitations. A range of outcomes were reported, yet some outcomes were assessed in only a single study, which may have affected the strength of the evidence presented in our results. Only one reviewer was involved in the search and selection of studies to be included; however, all search findings were discussed within the research team, including studies where eligibility was not clear (*n* = 4), and data extracted were checked twice by the lead author to ensure accuracy and interpretation. The search strategy was limited to articles published in the English language, which may have resulted in the exclusion of articles published in India’s vernacular languages. However, the authors do not believe this will have biased the results of this review, as evidence suggests that restricting reviews to English‐language articles does not affect the conclusions drawn for the majority of medical topics [46]. Finally, studies were excluded if they did not report offspring outcomes following live birth, which may lead to an underestimation of the adverse outcomes associated with maternal anemia.

The findings from this review highlight that anemia is a persisting issue for pregnant women in India, and much remains unclear about the effects anemia during pregnancy has on offspring. Further research is needed to identify to what extent, if any, adverse outcomes persist into childhood and adulthood. Most of the included studies (*n* = 13) have relatively short follow‐up periods (< 1 year), thus making it difficult to determine whether associations reported continue through childhood and into adulthood development stages. In addition, since only two studies had follow‐up periods longer than a year, the generalizability of their results is limited and thus highlights the need for further research focused on the long‐term effects of maternal anemia on offspring. Much focus has been given to anthropometry outcomes, i.e., child growth, yet gaps remain in terms of cognitive development. Further, there has been little effort to assess the underlying cause(s) of anemia using additional laboratory parameters and how differing causes and types of anemia affect offspring growth and development. We suggest that further research should focus on the long‐term follow‐up of offspring into early adulthood and that data collection should include cognitive development alongside physical growth. In addition, future studies should aim to use the WHO’s thresholds for anemia to allow for comparison across studies and for uniformity in the literature. Finally, to aid with achieving the WHO anemia targets, we suggest an appraisal of current anemia‐prevention strategies in India and for policymakers to consider more targeted prevention strategies for pregnant women that take into account trimester‐specific changes in iron demands and consider supplementation for other micronutrient deficiencies such as vitamin B12 and folate co‐supplementation.

## 5. Conclusion

This scoping review has highlighted that the reported prevalence of maternal anemia varies drastically across India and that maternal anemia during pregnancy may be a risk factor for adverse offspring physical and cognitive development. However, studies conducted in India have relatively short follow‐up periods. There is a need for further research to assess the long‐term effects of maternal anemia on child development to design and deliver suitable health interventions.

## Ethics Statement

Ethical approval and informed consent were not required for this scoping review.

## Conflicts of Interest

The authors declare no conflicts of interest.

## Author Contributions

Melissa T. Benavente: conception and design of the review; development of search strategy, screening, study selection, data charting, and data extraction; drafting the original article; approval of final manuscript. Nophar Geifman and Sarah Bath: review and editing the article; approval of final manuscript. Anand Ahankari: conception and design of the review; review and editing the article; approval of final manuscript.

## Funding

This review article was supported by the lead author’s PhD scholarship, awarded by the University of Surrey Doctoral College (Grant number TE4026).

## Supporting Information

Supporting Information 1. Search strategy.

Supporting Information 2. Definition of outcomes.

Supporting Information 3. Preferred Reporting Items for Systematic reviews and Meta‐Analyses extension for Scoping Reviews (PRISMA‐ScR) Checklist.

## Supporting information


**Supporting Information** Additional supporting information can be found online in the Supporting Information section.

## Data Availability

Data sharing is not applicable, as no datasets were generated or analyzed during this review.
