# Supplementary material for: Maternal Anemia and Offspring Outcomes in India: A Scoping Review
Source: Anemia. 2025 Oct 16;2025:2850956. doi: 10.1155/anem/2850956 (PMC12539666; doi:10.1155/anem/2850956)
Supplement: Supplementary file 1 — Supporting Information Additional supporting information can be found online in the Supporting Information section. [file ANEM-2025-2850956-s001.docx]

**Supporting Information 1**

The following electronic databases were searched: Scopus, MEDLINE (EBSCOhost), Embase (Ovid), and Web of Science Core Collection (consisting of Science Citation Index Expanded, Social Sciences Citation Index, Arts & Humanities Citation Index, Conference Proceedings Citation Index – Science, Conference Proceedings Index – Social Science & Humanities, Book Citation Index – Science, Book Citation Index – Social Sciences & Humanities, Emerging Sources Citation Index, Current Chemical Reactions, Index Chemicus). All searches were conducted on 23/01/2023 and, where applicable in the database, it was limited to English language and for studies published from 1990 onwards.

Reference lists of included articles (N=10) were manually screened to identify additional five studies (total included studies= 15).

MEDLINE (EBSCOhost)

1. AB (Iron OR iron deficiency OR iron deficiency an#emia OR an#emia) OR TI (Iron OR iron deficiency OR iron deficiency an#emia OR an#emia) OR (MH "Anemia") OR (MH "Anemia, Hypochromic") OR (MH "Anemia, Iron-Deficiency")

AND

1. AB (India OR Indian) OR TI (India OR Indian) OR (MH "India")

AND

1. AB (cohort study OR cohort OR cohort study design OR cohort design) OR TI (cohort study OR cohort OR cohort study design OR cohort design OR (MH "Cohort Studies") OR (MH "Longitudinal Studies") OR (MH "Epidemiologic Studies") OR (MH "Prospective Studies") OR (MH "Retrospective Studies") OR (MH "Follow-Up Studies")

AND

1. AB (offspring OR child* OR baby OR newborn OR adolescen*) OR TI (offspring OR child* OR baby OR newborn OR adolescen*) OR (MH "Child") OR (MH "Child Health") OR (MH "Child Poverty") OR (MH "Child, Preschool") OR (MH "Child Development") OR (MH "Child Nutrition Sciences") OR (MH "Parent-Child Relations") OR (MH "Child Nutrition Disorders") OR (MH "Maternal-Child Nursing") OR (MH "Child Nutritional Physiological Phenomena") OR (MH "Infant Health") or (MH "Adolescent") OR (MH "Adolescent Health") OR (MH "Adolescent Medicine") OR (MH "Adolescent Development") OR (MH "Child Nutrition Sciences") OR (MH "Pregnancy in Adolescence") OR (MH "Pediatrics") OR (MH "Child Development") OR (MH "Child Health")

AND

1. AB (pregnan* OR pregnant * OR maternal) OR TI (pregnan* OR pregnant * OR maternal) OR (MH "Pregnant Women") OR (MH "Pregnancy Complications, Hematologic") or (MH "Pregnancy") OR (MH "Pregnancy Trimesters") OR (MH "Pregnancy Outcome") OR (MH "Pregnancy Complications") OR (MH "Pregnancy, Multiple") OR (MH "Pregnancy Complications, Hematologic") OR (MH "Pregnancy in Adolescence") OR (MH "Pregnancy Trimester, First") OR (MH "Pregnancy Trimester, Second") OR (MH "Pregnancy Trimester, Third") OR (MH "Maternal Health") or (MH "Infant, Newborn") OR (MH "Infant, Newborn, Diseases") OR (MH "Live Birth") OR (MH "Infant, Small for Gestational Age") OR (MH "Infant, Low Birth Weight")
2. Limited to “human”, English language and between 01/01/1990-01/03/2023

Embase (OVID)

1. anemia/ or 'iron deficiency anemia'/ or 'microcytic anemia'/ or (Iron or 'iron deficiency' or 'iron deficiency an?emia' or an?emia).ab,ti,kw.
2. limit 1 to (human and english language and yr="1990 -Current")
3. india/ or indian/ or (India OR Indian).ab,ti,kw
4. limit 3 to (human and english language and yr="1990 -Current")
5. 'cohort analysis'/ or ('cohort study' OR cohort OR 'cohort study design' OR 'cohort design').ab,ti,kw.
6. limit 5 to (human and english language and yr="1990 -Current")
7. progeny/ OR 'child rearing'/ or 'preschool child'/ or 'maternal child health care'/ or 'child development'/ or 'father child relation'/ or 'child health'/ or 'child nutrition'/ or 'child growth'/ or child/ or 'mother child relation'/ or 'child parent relation'/ or 'child health care'/ or 'school child'/ or 'child care'/ OR baby/ OR 'newborn care'/ or "parameters concerning the fetus, newborn and pregnancy"/ or newborn/ or 'newborn disease'/ OR adolescence/ or adolescent/ or (offspring OR child* OR baby OR newborn OR adolescen*).ab,ti,kw.
8. limit 7 to (human and english language and yr="1990 -Current")
9. pregnant woman/ or pregnancy/ or (pregnan* OR 'pregnant wom#n' OR maternal).ab,ti,kw.
10. limit 9 to (human and english language and yr="1990 -Current")
11. 2 and 4 and 6 and 8 and 10

Web of Science

1. (Iron OR "iron deficiency" OR "iron deficiency an*emia" OR an*emia OR microcytic an*emia OR hypochromic an*emia)

AND

1. (India OR Indian)

AND

1. (“cohort study” OR cohort OR "cohort study design" OR "cohort design" OR “prospective study” OR “retrospective study” OR “longitudinal study”)

AND

1. (offspring OR child* OR baby OR newborn OR adolescen* OR infan* OR progeny)

AND

1. (pregnancy OR pregnant OR pregnant wom?n OR maternal)
2. Limited to “topic” and 01/01/1990-31/12/2023

Scopus

1. “iron deficiency” OR "iron deficiency anemia" OR "iron deficiency anaemia" OR anemia OR anaemia OR “microcytic anemia” OR “microcytic anaemia” OR “hypochromic anemia” OR “hypochromic anaemia”

AND

1. India OR Indian

AND

1. “cohort study” OR cohort OR "cohort study design" OR "cohort design" OR “prospective study” OR “retrospective study” OR “longitudinal study”

AND

1. 4. offspring OR child* OR baby OR newborn OR adolescen*OR infan* OR progeny

AND

1. 5. pregnan* OR "pregnant wom?n" OR maternal

**Supporting Information 2 (Table S2)**

| **Outcome** | **Definition /unit of measure** |
| --- | --- |
| Anthropometry |  |
| Low birth weight | < 2500 g /< 2.5 Kg |
| Birth weight percentile | Calculated using Lubchenco’s growth chart^1^ |
| Z-scores |  |
| Weight-for-age, length-for-age, weight-for-length | Estimated using either the WHO Growth Standards^2^ or Centers for Disease Control and Prevention Growth Charts (CDC)^3^.  Z-scores which are 2 standard deviations (SD) below the respective WAZ, LAZ, WLZ are defined as underweight, stunting, and wasting, respectively. |
| BMI-for-age | Estimated using WHO Growth Standards^2^  Z-scores which are 2 SD below the respective BMI-for-age are defined as thinness |
| Head circumference-for-age | Estimated using CDC Growth Charts^3^. |
| Apgar score | Assessment of offspring colour, heart rate, reflexes, muscle tone, and respiration. Each component is given a score between 0 and 2. Assessment taken 1 minute and 5 minutes after birth^4^. |
| Gestational age | Confirmed using an early dating ultrasound. Or calculated based on last menstrual period only, or alongside New Ballad Score after birth^5^. |
| Small for gestational age | Defined as birth weight less than the 10^th^ percentile of gestational age based on INTERGROWTH 21^st 6^, or defined as birth weight less than 3^rd^ percentile for gestation^7^. |
| Cognitive skills |  |
| FREDI 0-3 | Adapted version of the child development assessment FREDI 0-3^8^ to improve suitability to the Bihar context. Consists of a child assessment and parent questionnaires on offspring: fine and gross motor development, cognition, socioemotional development, receptive and expressive language development. Presented as z-scores. |
| Neonatal Behavioural Assessment Score | Assessment of offspring capacity to respond to external manipulations. Consists of 28 behavioural items^9^ and 18 reflex items which were reduced to seven behaviour dimensions, according to a data reduction scheme^10^: abnormal reflexes, motor maturity, habituation, regulation of state, range of state, orientation, and autonomic stability. |
| ^1^ Lubchenco L O, Hansman C, Dressler M, Boyd E. Intrauterine growth as estimated from liveborn birth-weight data at 24 to 42 weeks of gestation. Pediatrics. 1963;32:793–800.  ^2^ Department of Nutrition for Health and Development. WHO Child Growth Standards [Internet]. WHO; 2006.  ^3^ Centres for Disease Control and Prevention. 2000 CDC Growth Charts for the United States: Methods and Development [Internet]. National Centre for Health Statistics; May 20002.  ^4^ AMERICAN ACADEMY OF PEDIATRICS COMMITTEE ON FETUS AND NEWBORN, AMERICAN COLLEGE OF OBSTETRICIANS AND GYNECOLOGISTS COMMITTEE ON OBSTETRIC PRACTICE, Watterberg KL, Aucott S, Benitz WE, Cummings JJ, et al. The Apgar Score. Pediatrics. 2015 Oct 1;136(4):819–22.  ^5^ Ballard JL, Khoury JC, Wedig K, Wang L, Eilers-Walsman BL, Lipp R. New Ballard Score, expanded to include extremely premature infants. J Pediatr. 1991 Sep;119(3):417–23.  ^6^ Villar J, Ismail LC, Victora CG, Ohuma EO, Bertino E, Altman DG, et al. International standards for newborn weight, length, and head circumference by gestational age and sex: the Newborn Cross-Sectional Study of the INTERGROWTH-21st Project. The Lancet. 2014 Sep;384(9946):857–68.  ^7^ Bora R, Sable C, Wolfson J, Boro K, Rao R. Prevalence of anemia in pregnant women and its effect on neonatal outcomes in Northeast India. J Matern Fetal Neonatal Med. 2014 Jun;27(9):887–91.  ^8^ Hasselhorn M. FREDI 0–3. Frühe Bild. 2017;6:101–3.  ^9^ Brazelton TB. Neonatal behavioral assessment scale. London : Philadelphia: Spastics International Medical Publications : Heinemann Medical ; J. B. Lippincott; 1973. 66 p. (Clinics in developmental medicine ; no. 50).  ^10^ Lester. Regional obstetric anesthesia and newborn behavior: a reanalysis toward synergistic effects. Child Dev. 1982;53(3):687–92. | |

**Supporting Information 3 (Table S3)**

| **SECTION** | **ITEM** | **PRISMA-ScR CHECKLIST ITEM** | **REPORTED ON PAGE #** |
| --- | --- | --- | --- |
| **TITLE** | | | |
| Title | 1 | Identify the report as a scoping review. | 1 |
| **ABSTRACT** | | | |
| Structured summary | 2 | Provide a structured summary that includes (as applicable): background, objectives, eligibility criteria, sources of evidence, charting methods, results, and conclusions that relate to the review questions and objectives. | 2 |
| **INTRODUCTION** | | | |
| Rationale | 3 | Describe the rationale for the review in the context of what is already known. Explain why the review questions/objectives lend themselves to a scoping review approach. | 2-4 |
| Objectives | 4 | Provide an explicit statement of the questions and objectives being addressed with reference to their key elements (e.g., population or participants, concepts, and context) or other relevant key elements used to conceptualize the review questions and/or objectives. | 4 |
| **METHODS** | | | |
| Protocol and registration | 5 | Indicate whether a review protocol exists; state if and where it can be accessed (e.g., a Web address); and if available, provide registration information, including the registration number. | Not done |
| Eligibility criteria | 6 | Specify characteristics of the sources of evidence used as eligibility criteria (e.g., years considered, language, and publication status), and provide a rationale. | Figure 2 |
| Information sources* | 7 | Describe all information sources in the search (e.g., databases with dates of coverage and contact with authors to identify additional sources), as well as the date the most recent search was executed. | 5 & S1 |
| Search | 8 | Present the full electronic search strategy for at least 1 database, including any limits used, such that it could be repeated. | S1 |
| Selection of sources of evidence† | 9 | State the process for selecting sources of evidence (i.e., screening and eligibility) included in the scoping review. | 5 |
| Data charting process‡ | 10 | Describe the methods of charting data from the included sources of evidence (e.g., calibrated forms or forms that have been tested by the team before their use, and whether data charting was done independently or in duplicate) and any processes for obtaining and confirming data from investigators. | 5 |
| Data items | 11 | List and define all variables for which data were sought and any assumptions and simplifications made. | S2 |
| Critical appraisal of individual sources of evidence§ | 12 | If done, provide a rationale for conducting a critical appraisal of included sources of evidence; describe the methods used and how this information was used in any data synthesis (if appropriate). | Not done |
| Synthesis of results | 13 | Describe the methods of handling and summarizing the data that were charted. | 5 |
| **RESULTS** | | | |
| Selection of sources of evidence | 14 | Give numbers of sources of evidence screened, assessed for eligibility, and included in the review, with reasons for exclusions at each stage, ideally using a flow diagram. | Figure 1 |
| Characteristics of sources of evidence | 15 | For each source of evidence, present characteristics for which data were charted and provide the citations. | Table 2 |
| Critical appraisal within sources of evidence | 16 | If done, present data on critical appraisal of included sources of evidence (see item 12). | Not done |
| Results of individual sources of evidence | 17 | For each included source of evidence, present the relevant data that were charted that relate to the review questions and objectives. | Table 3 |
| Synthesis of results | 18 | Summarize and/or present the charting results as they relate to the review questions and objectives. | 6-12 |
| **DISCUSSION** | | | |
| Summary of evidence | 19 | Summarize the main results (including an overview of concepts, themes, and types of evidence available), link to the review questions and objectives, and consider the relevance to key groups. | 12-16 |
| Limitations | 20 | Discuss the limitations of the scoping review process. | 15 |
| Conclusions | 21 | Provide a general interpretation of the results with respect to the review questions and objectives, as well as potential implications and/or next steps. | 16 |
| **FUNDING** | | | |
| Funding | 22 | Describe sources of funding for the included sources of evidence, as well as sources of funding for the scoping review. Describe the role of the funders of the scoping review. | 16 |

JBI = Joanna Briggs Institute; PRISMA-ScR = Preferred Reporting Items for Systematic reviews and Meta-Analyses extension for Scoping Reviews.

* Where *sources of evidence* (see second footnote) are compiled from, such as bibliographic databases, social media platforms, and Web sites.

† A more inclusive/heterogeneous term used to account for the different types of evidence or data sources (e.g., quantitative and/or qualitative research, expert opinion, and policy documents) that may be eligible in a scoping review as opposed to only studies. This is not to be confused with *information sources* (see first footnote).

‡ The frameworks by Arksey and O’Malley (6) and Levac and colleagues (7) and the JBI guidance (4, 5) refer to the process of data extraction in a scoping review as data charting*.*

§ The process of systematically examining research evidence to assess its validity, results, and relevance before using it to inform a decision. This term is used for items 12 and 19 instead of "risk of bias" (which is more applicable to systematic reviews of interventions) to include and acknowledge the various sources of evidence that may be used in a scoping review (e.g., quantitative and/or qualitative research, expert opinion, and policy document).

*From:* Tricco AC, Lillie E, Zarin W, O'Brien KK, Colquhoun H, Levac D, et al. PRISMA Extension for Scoping Reviews (PRISMAScR): Checklist and Explanation. Ann Intern Med. 2018;169:467–473. [doi: 10.7326/M18-0850](http://annals.org/aim/fullarticle/2700389/prisma-extension-scoping-reviews-prisma-scr-checklist-explanation).
